# Amyloidogenic 60–71 deletion/ValThr insertion mutation of apolipoprotein A-I generates a new aggregation-prone segment that promotes nucleation through entropic effects

**DOI:** 10.1038/s41598-023-45803-y

**Published:** 2023-10-28

**Authors:** Norihiro Namba, Takashi Ohgita, Hiroko Tamagaki-Asahina, Kazuchika Nishitsuji, Toshinori Shimanouchi, Takeshi Sato, Hiroyuki Saito

**Affiliations:** 1 Laboratory of Biophysical Chemistry, Kyoto Pharmaceutical University, 5 Misasagi-Nakauchi-cho, Yamashina-ku, Kyoto, 607-8414 Japan; 2 Division of Liberal Arts Sciences, Kyoto Pharmaceutical University, 5 Misasagi-Nakauchi-cho, Yamashina-ku, Kyoto, 607-8414 Japan; 3 Department of Biochemistry, Wakayama Medical University, 811-1 Kimiidera, Wakayama, 641-8509 Japan; 4 Graduate School of Environmental and Life Science, Okayama University, Okayama, 700-8530 Japan

**Keywords:** Protein aggregation, Biophysical chemistry

## Abstract

The N-terminal fragment of apolipoprotein A-I (apoA-I), comprising residues 1–83, contains three segments prone to aggregation: residues 14–22, 53–58, and 67–72. We previously demonstrated that residues 14–22 are critical in apoA-I fibril formation while residues 53–58 entropically drove the nucleation process. Here, we investigated the impact of amyloidogenic mutations (Δ60–71/VT, Δ70–72, and F71Y) located around residues 67–72 on fibril formation by the apoA-I 1–83 fragment. Thioflavin T fluorescence assay demonstrated that the Δ60–71/VT mutation significantly enhances both nucleation and fibril elongation rates, whereas the Δ70–72 and F71Y mutations had minimal effects. Circular dichroism measurements and microscopic observations revealed that all variant fragments formed straight fibrils, transitioning from random coils to β-sheet structures. Kinetic analysis demonstrated that primary nucleation is the dominant step in fibril formation, with fibril elongation reaching saturation at high protein concentrations. Thermodynamically, both nucleation and fibril elongation were enthalpically and entropically unfavorable in all apoA-I 1–83 variants, in which the entropic barrier of nucleation was almost eliminated for the Δ60–71/VT variant. Taken together, our results suggest the presence of new aggregation-prone segment in the Δ60–71/VT variant that promotes nucleation through entropic effects.

## Introduction

Apolipoprotein A-I (apoA-I), the major constituent of high-density lipoprotein (HDL) in plasma, is essential for the generation and maturation of HDL particles^1,2^. The human apoA-I is a 243-residue protein consisting of an N-terminal α-helix bundle region spanning residues 1–189 and a separate C-terminal region that is disordered and spans the remaining portion of the molecule^3–5^. Although apoA-I is primarily associated with the surface of HDL, approximately 5–10% of apoA-I transiently dissociates from HDL in a lipid-poor or lipid-free state^6,7^. This lipid-poor/free apoA-I is known to play a crucial role in facilitating the ATP-binding cassette A1-mediated efflux of cellular phospholipids and cholesterol, thereby promoting the formation of nascent HDL particles^8,9^. Conversely, the structurally labile lipid-free apoA-I is believed to be a precursor of amyloid fibrils^10,11^. Specific amyloidogenic mutations have been suggested to decrease the stability and lipid affinity of apoA-I^12–14^, consequently shifting the distribution from HDL-bound to lipid-poor/free apoA-I^10^.

Amyloidogenic mutations of apoA-I cause hereditary apoA-I amyloidosis which is characterized by the extracellular deposition of amyloid fibrils formed by N-terminal 9–11 kDa fragments of variant proteins in specific organs, such as the heart, liver, kidneys, and gastrointestinal tract^15,16^. To date, more than 20 amyloidogenic mutations in human apoA-I have been identified, primarily concentrated in two regions located within the N-terminal domain: residues 26–107 and 154–178^11,17^. Sequence analyses have predicted the presence of three aggregation-prone segments within the N-terminal residues 1–100 of apoA-I: residues 14–22, 53–58, and 67–72^18–20^. It has been consistently observed that the N-terminal 1–83 or 1–93 fragments of apoA-I exhibit a strong propensity to form amyloid fibrils^12,21,22^, and synthetic apoA-I fragment peptides containing either the first (residues 14‒22) or second (residues 53‒58) aggregation-prone segment can form amyloid fibrils with a transition to the β-structure^23,24^. Recently, we conducted experiments with a series of deletion variants, which demonstrated the crucial roles of the two amyloidogenic segments (residues 14–22 and 53–58) in fibril formation by the N-terminal 1–83 fragment of apoA-I: residues 14‒22 are crucial for β-transition and fibril formation, while residues 53‒58 entropically drive the nucleation step in fibril formation^25^.

In this study, we investigated the impact of naturally occurring amyloidogenic mutations Δ60–71/ValThr insertion (Δ60–71/VT)^26^, Δ70–72^27^, and F71Y^28^ occurring around the third aggregation-prone segment (residues 67–72) on the fibril-forming properties of the N-terminal 1–83 fragment of apoA-I. Our kinetic and thermodynamic analyses revealed that the Δ60–71/VT mutation generates a new aggregation-prone segment around residues 53–62, which promotes the nucleation process through reducing entropic barrier, thereby facilitating the aggregation and fibril formation of the apoA-I 1–83 fragment.

## Results

### Effects of amyloidogenic mutations of structure and fibril-forming properties of full-length apoA-I

**Figure 1:**
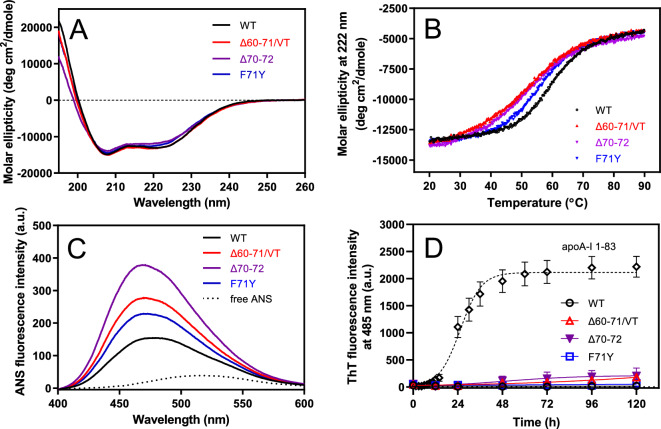
Effects of amyloidogenic mutations on the structural stability and fibril-forming propensity of full-length apoA-I. (**A**) CD spectra of apoA-I wild-type (WT), Δ60‒71/VT, Δ70‒72, and F71Y. (**B**) Thermal unfolding of apoA-I variants monitored by the ellipticity at 222 nm. (**C**) ANS fluorescence spectra in the presence of apoA-I variants. ANS fluorescence spectrum of free ANS in buffer was shown for comparison. *a. u.*, arbitrary units. (**D**) ThT fluorescence intensity for apoA-I WT (open circle), Δ60‒71/VT (open triangle), Δ70‒72 (filled reverse triangle), and F71Y (open square) were plotted as a function of time. The data for apoA-I 1–83 fragment (open diamond) was shown for comparison. Protein and ThT concentrations were 200 μg/ml and 10 μM, respectively. *a. u.*, arbitrary units.

We initially examined the effects of the Δ60–71/VT, Δ70–72, and F71Y mutations on the structure and stability of full-length apoA-I. Circular dichroism (CD) spectra indicated that all apoA-I variants exhibited similar α-helical structures (Fig. 1A). However, thermal unfolding measurements, monitored by ellipticity at 222 nm, demonstrated a decrease in the thermal stability of apoA-I in the amyloidogenic variants, particularly in Δ60–71/VT and Δ70–72 (Fig. 1B and Table 1). Consistently, 8-anilino-1-naphthalenesulfonic acid (ANS) fluorescence spectra, which reflect the exposure of hydrophobic surfaces of proteins^29^, exhibited a significant increase in amyloidogenic variants in the following order of Δ70–72 > Δ60–71/VT > F71Y (Fig. 1C and Table 1). These findings indicate that the amyloidogenic variants, particularly Δ60–71/VT and Δ70–72, considerably destabilize the N-terminal α-helix bundle structure of apoA-I^3,12^. A thioflavin T (ThT) fluorescence assay demonstrated that none of the full-length amyloidogenic variants exhibited a propensity to form amyloid fibrils, similar to the N-terminal 1**‒**83 fragment of apoA-I (Fig. 1D). This indicates that the destabilization of the protein structure by amyloidogenic mutations does not promote apoA-I fibril formation at neutral pH^12^.

**Table 1:** α-Helix content, thermal denaturation parameters, and ANS binding for full-length apoA-I variants.

	α-helix^a^ (%)	Thermal denaturation			ANS fluorescence intensity^e^
		T_m_^b^ (°C)	Cooperativity index^c^	ΔH_V_^d^ (kJ/mol)	
ApoA-I wild-type	41 ± 2	60	8.9	130	1.0
Δ60‒71/VT	41 ± 1	52	5.0	92	1.7
Δ70‒72	41 ± 3	51	5.3	97	2.3
F71Y	41 ± 4	56	7.7	121	1.4

^a^Mean ± SD from at least three independent experiments.

^b^The reproducibility in *T*_m_ is ± 1.3 ºC.

^c^Calculated as described under “Materials and methods”.

^d^Estimated error is within ± 4 kJ/mol.

^e^Values are ratios of wild-type apoA-I. Estimated error is within ± 0.1.

### Comparison of the fibril-forming properties of apoA-I 1‒83 variants

**Figure 2:**
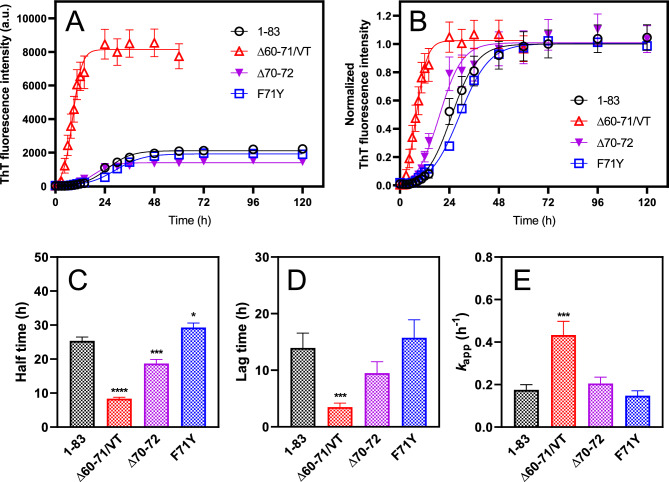
Kinetics of aggregation of apoA-I 1‒83 and amyloidogenic variants were monitored by ThT fluorescence at 37 °C. (**A**,**B**) ThT fluorescence intensity (**A**) or normalized to maximum ThT fluorescence (**B**) were plotted as a function of time. (open circle) apoA-I 1‒83; (open triangle) apoA-I 1‒83 Δ60‒71/VT; (filled reverse triangle) apoA-I 1‒83 Δ70‒72; (open square) apoA-I 1‒83 F71Y. The data were from at least three independent experiments. The solid lines are the fitted curves by the sigmoidal Eq. 1. Protein and ThT concentrations were 200 μg/ml and 10 μM, respectively. *a. u.*, arbitrary units. (**C–E**) Comparison of half time (**C**), lag time (**D**), and apparent rate constant (**E**) for the growth of the fibrils of apoA-I 1‒83 variants according to the sigmoidal equation. ^*^, *p* < 0.05; ^***^, *p* < 0.001; ^****^, *p* < 0.0001 versus “apoA-I 1‒83”.

Next, we assessed the effects of amyloidogenic mutations on the fibril-forming propensities of the 1**‒**83 fragment of apoA-I. Among the amyloidogenic variants, the Δ60–71/VT variant of apoA-I 1–83 fragment exhibited significantly enhanced ThT fluorescence intensity compared to the 1**‒**83 fragment (Fig. 2A, B). A comparison of the half time at which half of the monomer protein is converted to the fibrillar form indicated that the Δ60–71/VT and Δ70–72 mutations significantly enhance the amyloid fibril formation of apoA-I 1–83 (Fig. 2C). Kinetic analysis based on fitting to the empirical sigmoidal equation demonstrated that the Δ60–71/VT mutation significantly reduces the lag time and increases the apparent rate constant for fibril elongation compared to the 1–83 fragment (Fig. 2D, E). Kinetic analysis according to the Finke-Watzky two-step model, in which homogeneous nucleation is followed by autocatalytic heterogeneous fibril elongation^30,31^ provided similar conclusions: the Δ60–71/VT mutation significantly increases both the rate constants of nucleation and fibril elongation in fibril formation (Supplementary Fig. S1).

**Figure 3:**
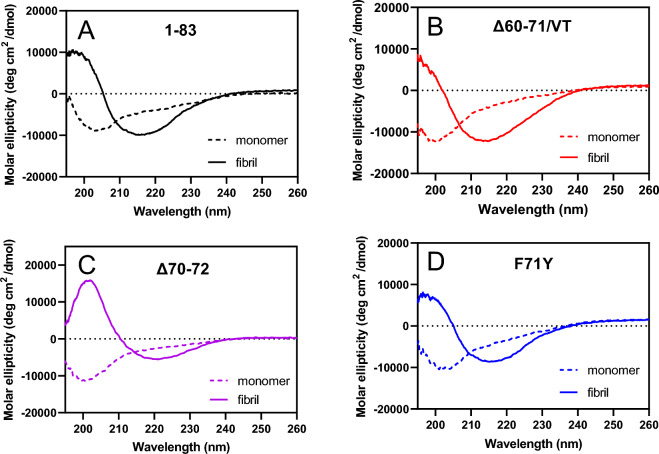
CD spectra of apoA-I 1‒83 and amyloidogenic variants in the monomer (before incubation, dashed line) and fibrillar (after 120 h incubation, solid line) forms. (**A**) apoA-I 1‒83, (**B**) apoA-I 1‒83 Δ60‒71/VT, (**C**) apoA-I 1‒83 Δ70‒72, (**D**) apoA-I 1‒83 F71Y. Protein concentration was 50 μg/ml.

**Figure 4:**
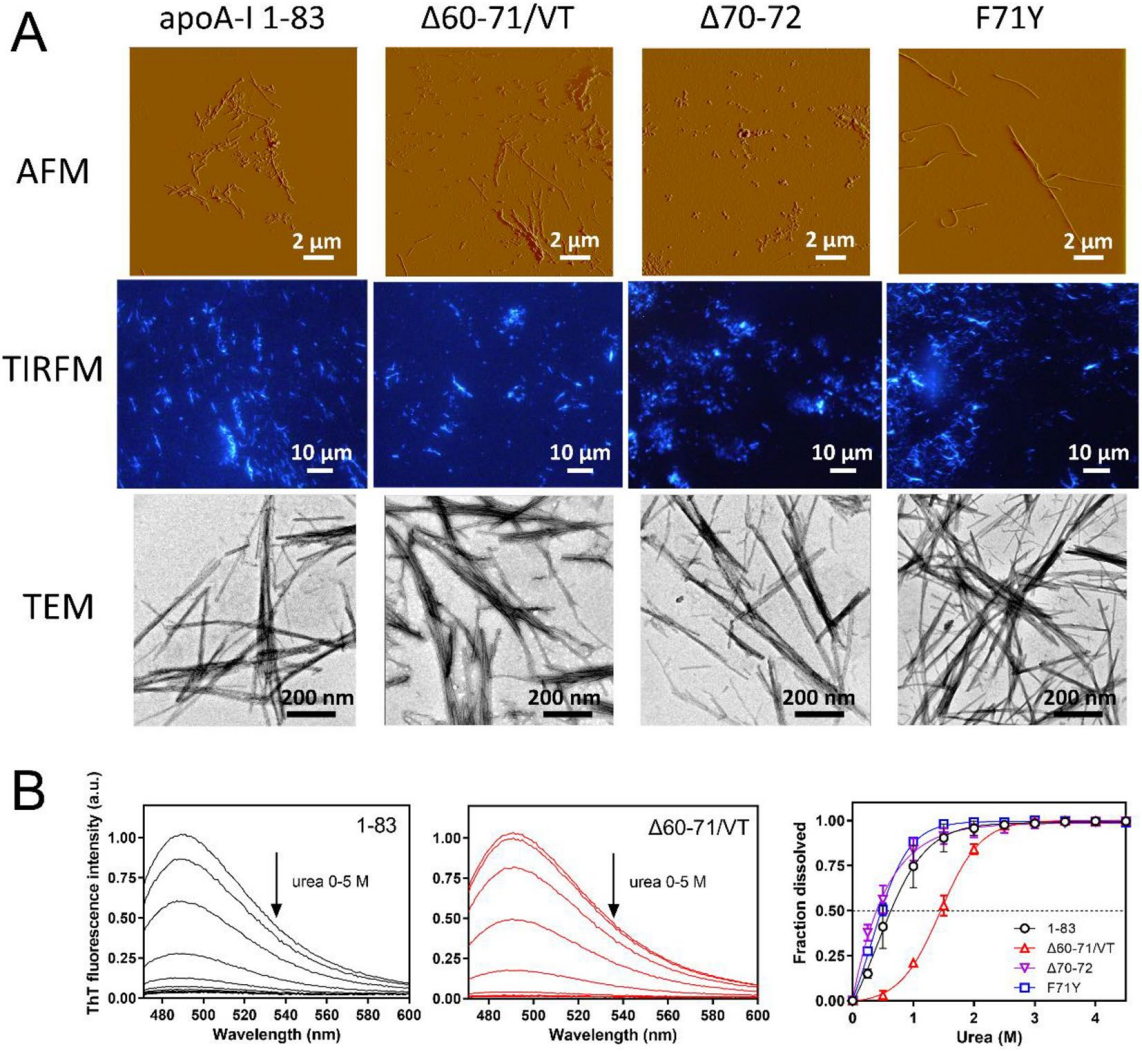
Structural morphology and stability of fibrils formed by apoA-I 1‒83 and amyloidogenic variants. (**A**) AFM, TIRFM, and TEM images of apoA-I 1‒83 variants after 120 h incubation. Scale bars represent 2 μm (AFM), 10 μm (TIRFM), and 200 nm (TEM), respectively. (**B**) Urea-induced disaggregation of fibrils formed by apoA-I 1‒83 variants. Changes in ThT fluorescence spectra of apoA-I 1‒83 (left) and apoA-I 1‒83 Δ60‒71/VT (center) with increasing concentrations of urea. *a. u.*, arbitrary units. Comparison of urea-induced disaggregation curves for fibrils formed by apoA-I 1‒83 variants (right).

Secondary structural changes in the 1–83 variants during incubation were assessed using CD measurements. As shown in Fig. 3, all 1–83 variants, which existed as random coil structures before incubation, exhibited single minimum spectra at approximately 216 nm after incubation for 120 h, implying conversion to a β-sheet-rich structure. Atomic force microscopy (AFM), total internal reflection fluorescence microscopy (TIRFM), and transmission electron microscopy (TEM) revealed that all the 1–83 variants formed ThT reactive thin and straight fibrils after incubation (Fig. 4A). However, despite the similar apparent morphology of fibrils formed by the amyloidogenic variants, the fibrils of the Δ60–71/VT variant exhibited higher stability against urea-induced disaggregation than the other variants (Fig. 4B and Supplementary Fig. S2). This suggests the presence of differences in the β-sheet structure of the Δ60–71/VT variant. In addition, the fibrils formed by all amyloidogenic 1–83 variants and the wild-type fragment induced similar cytotoxicity in HEK293 cells (Supplementary Fig. S3), which is consistent with previous findings that the formation of fibril structures is essential for the cytotoxicity of apoA-I 1–83 variants^12,25^.

### Effects of monomer concentration and seed fibrils on fibril-forming kinetics of apoA-I 1‒83 variants

**Figure 5:**
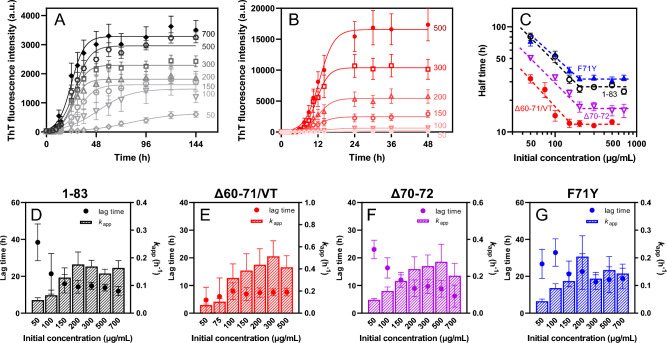
Effect of initial monomer concentration on aggregation kinetics of apoA-I 1‒83 and amyloidogenic variants at 37 °C. (**A**,**B**) Kinetics of formation of amyloid fibrils monitored by ThT fluorescence for apoA-I 1‒83 (**A**) and apoA-I 1‒83 Δ60‒71/VT (B) with increasing initial monomer concentrations. ThT concentration was 10 μM. *a. u.*, arbitrary units. (**C**) The power-law relationships between the half time and the initial monomer concentration. (open circle) apoA-I 1‒83; (filled circle) apoA-I 1‒83 Δ60‒71/VT; (open inverse triangle) apoA-I 1‒83 Δ70‒72; (filled triangle) apoA-I 1‒83 F71Y. (**D**–**G**) Effects of initial monomer concentration on lag time and apparent rate constant for the growth of fibrils for apoA-I 1‒83 (**D**), apoA-I 1‒83 Δ60‒71/VT (**E**), apoA-I 1‒83 Δ70‒72 (**F**), and apoA-I 1‒83 F71Y (**G**).

Next, we investigated the dependence of fibril-forming kinetics on the monomer concentration of the apoA-I 1–83 variants. Figure 5A, B display the time courses of ThT fluorescence intensities for the apoA-I 1–83 and Δ60–71/VT variants at various initial monomer concentrations. Previous reports have demonstrated that the half time of fibril formation depends on the initial monomer concentration, indicating that either nucleation or fibril elongation is the dominant step^32,33^. The half time versus initial monomer concentration plots for the apoA-I 1–83 variants, presented as double logarithmic graphs, reveal a linear decrease in half time at low monomer concentrations, while the slope becomes flat at high monomer concentrations (Fig. 5C). This monomer concentration-independent behavior at the half time suggests the saturation of the elongation step at high monomer concentrations^33,34^. The apparent rate constants for fibril elongation increase at low monomer concentrations but remain unchanged at high monomer concentrations for all 1–83 variants (Fig. 5D–G). In contrast, the lag time tends to decrease with increasing monomer concentration, except for the Δ60–71/VT variant, where the lag time remains constant across all examined concentrations (Fig. 5D–G).

We also applied the online Amylofit program (https://www.amylofit.ch.cam.ac.uk)^32^ to the kinetic data shown in Fig. 5A, B with a model involving saturating elongation and secondary nucleation. From the double logarithmic plot of half time and initial monomer concentration, which gives the scaling exponent, γ, as the slop of the plot, we calculated the reaction order of secondary nucleation, *n*_2_, and was kept constant in the fitting process, while other kinetic parameters were allowed to vary. Best fitted results were shown in Fig. S4 in the Supplementary information. From the combined kinetic parameters for primary nucleation and elongation (*k*_n_*k*_+_) or secondary nucleation and elongation (*k*_2_*k*_+_), we obtained the effective noncatalytic fibril proliferation rates through primary and secondary processes, *λ* and *κ*, respectively. Given the relative magnitude of *λ* and *κ* determines the dominant process in the overall aggregation^35^, the finding of significant lower values *κ* compared *λ* indicates that the primary nucleation and elongation process is dominant over the secondary processes in apoA-I fibril formation.

**Figure 6:**
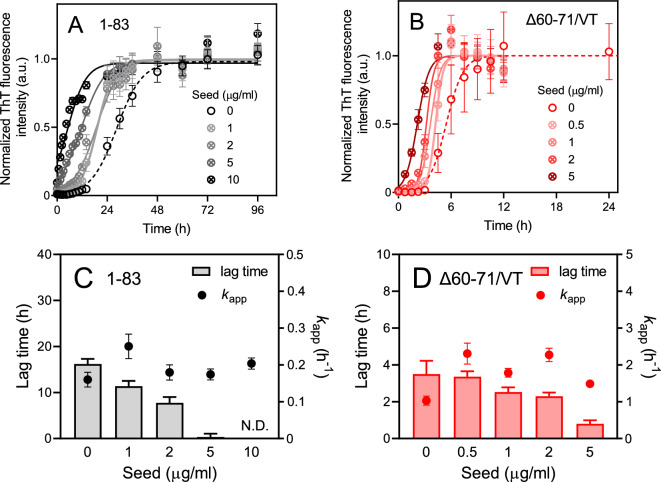
Effect of seed concentration on aggregation kinetics of apoA-I 1‒83 variants at 37 °C. (**A**,**B**) Kinetics of formation of amyloid fibrils monitored by ThT fluorescence for apoA-I 1‒83 (**A**) and apoA-I 1‒83 Δ60‒71/VT (**B**). Protein and ThT concentrations were 200 μg/ml and 10 μM, respectively. *a. u.*, arbitrary units. (**C**,**D**) Effects of seed concentration on lag time and apparent rate constant for the growth of fibrils for apoA-I 1‒83 (**C**) and apoA-I 1‒83 Δ60‒71/VT (**D**).

We further investigated the impact of preformed seeds on the kinetics of fibril formation for apoA-I 1–83 variants. It is widely recognized that the presence of preformed seeds strongly accelerates fibril formation by bypassing the conversion of soluble proteins into amyloid nuclei^35–37^. As depicted in Fig. 6A, B, the intensity of ThT fluorescence increased rapidly for the 1–83 and Δ60–71/VT variants with increasing concentrations of seed fibrils. Moreover, an increase in seed concentration gradually reduced the lag time without significantly affecting the apparent rate constant of fibril elongation, ultimately leading to the near elimination of the lag time beyond 5 μg/ml of seeds (Fig. 6C, D). This observation suggests that primary nucleation is the predominant process during the lag phase of fibril formation in the apoA-I 1–83 variants^35^.

### Thermodynamic analysis of fibril formation of apoA-I 1‒83 variants

**Figure 7:**
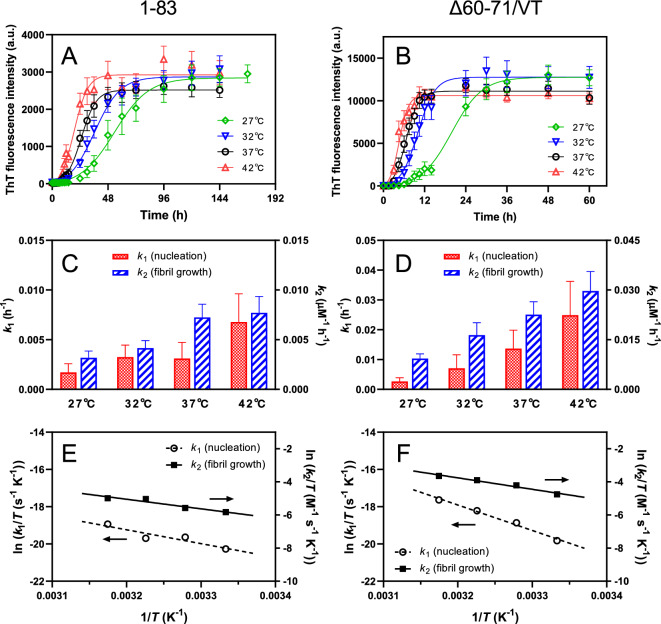
Thermodynamic analysis of the amyloid fibril formation of apoA-I 1‒83 variants. (**A**,**B**) Kinetics of amyloid fibril formation monitored by ThT fluorescence for apoA-I 1‒83 (**A**) and apoA-I 1‒83 Δ60‒71/VT (**B**) at different temperatures. The data were from at least three independent experiments. The solid lines are the fitted curves by the Finke-Watzky equation. Protein concentration was 200 μg/ml. *a. u.*, arbitrary units. (**C**,**D**) Comparison of the rate constants of nucleation (*k*_1_) and fibril growth (*k*_2_) for the fibril formation of apoA-I 1‒83 (**C**) and apoA-I 1‒83 Δ60‒71/VT (**D**). (**E**,**F**) Eyring plots of rate constants of *k*_1_ and *k*_2_ for the amyloid fibril formation of apoA-I 1‒83 (**E**) and apoA-I 1‒83 Δ60‒71/VT (**F**).

We conducted a thermodynamic analysis of the fibril-forming characteristics of apoA-I 1–83 variants by monitoring the kinetics of fibril formation at various temperatures^25,38,39^. We note that there are no secondary structural changes across the temperature range at which the thermodynamic analysis was performed^25^. Figure 7A shows the time course of ThT fluorescence intensities for apoA-I 1–83, with the curves fitted using the Finke-Watzky equation at different temperatures. The obtained rate constants for nucleation (*k*_1_) and fibril elongation (*k*_2_) increased as the temperature rose (Fig. 7C). Based on the linear plots derived from the Eyring equation for each rate constants of *k*_1_ and *k*_2_ (Fig. 7E), we determined the activation enthalpy (ΔH^*^) and entropy (ΔS^*^) for the nucleation and fibril elongation steps in fibril formation by apoA-I 1–83. As shown in Table 2, a comparison of the ΔH^*^ and ΔS^*^ values together with the activation Gibbs free energy (ΔG^*^) values demonstrated that both the nucleation and fibril elongation steps in the fibril formation of apoA-I 1–83 were enthalpically and entropically unfavorable.

**Table 2:** Thermodynamic parameters for nucleation and fibril growth in the fibril formation of apoA-I 1‒83 variants.

	∆H* (kJ/mol)^a^	∆S* (J/mol K)^a^	∆G* (kJ/mol)^b^
ApoA-I 1‒83			
Nucleation (k_1_)	62 ± 17	‒159 ± 56^d^	111
Fibril growth (k_2_)	48 ± 10	‒175 ± 32	102
∆60–71/VT			
Nucleation (k_1_)	114 ± 9^c^	17 ± 28	108
Fibril growth (k_2_)	57 ± 7^c^	‒134 ± 22^c^	99
∆70–72			
Nucleation (k_1_)	64 ± 23^c^	‒149 ± 71^d^	110
Fibril growth (k_2_)	73 ± 17^c^	‒92 ± 56^c^	102
F71Y			
Nucleation (k_1_)	83 ± 24^c^	‒92 ± 70^d^	111
Fibril growth (k_2_)	55 ± 13^c^	‒154 ± 42^c^	103

The data were from at least three independent experiments.

^a^ΔH* and ΔS* were obtained from the slope and y-intercept of the linear plot, respectively, according to Eq. (3).

^b^ΔG* was calculated from ΔH* and ΔS* according to ∆G* = ∆H* – *T*∆S* at 37 °C.

^c^Not significant versus apoA-I 1‒83.

^d^No significance among unfavorable entropies.

Similarly, amyloidogenic Δ60–71/VT, Δ70–72, and F71Y variants of apoA-I 1–83 exhibited temperature-dependent increases in ThT fluorescence intensity (Fig. 7B and Supplementary Fig. S5A, B). The Eyring plots of the rate constants *k*_1_ and *k*_2_ (Fig. 7F and Supplementary Fig. S5C, D) provided the thermodynamic parameters for each variant (Table 2). In sharp contrast to the wild-type apoA-I 1–83, the Δ60–71/VT variant showed a significant reduction in the unfavorable activation entropy and a concomitant increase in the unfavorable enthalpy for nucleation in fibril formation. For fibril elongation, the unfavorable activation enthalpy and entropy values for the Δ60–71/VT variant were similar to those of the wild-type apoA-I 1–83. These results indicate that the Δ60–71/VT mutation promoted fibril formation in apoA-I 1–83 through reducing entropic barrier for the nucleation process. As for the Δ70–72 and F71Y variants, similarly unfavorable activation enthalpy and entropy values were observed for both nucleation and fibril elongation, compared to those of the wild-type apoA-I 1–83. We note that the constancy of the ΔG^*^ values for the nucleation and fibril elongation of all the variants indicates the occurrence of the enthalpy–entropy compensation effects^40–42^.

### Effect of Δ60–71/VT mutation on the fibril-forming properties of the apoA-I 50‒75 peptide

The N-terminal residues 1–83 of apoA-I contained three aggregation-prone segments: residues 14–22, 53–58, and 67–72 (Supplementary Fig. S6A, B). Comparing the amyloid propensity prediction of amyloidogenic apoA-I 1–83 variants, we found that the Δ60–71/VT mutation combines two aggregation-prone segments, residues 53–58 and 67–72, to form a large aggregation-prone segment around residues 53–62. This newly formed segment exhibits amyloid propensity comparable to that of residues 14–22 (Supplementary Fig. S6C–E). To further evaluate the effects of the Δ60–71/VT mutation on the fibril-forming propensity of the N-terminal residues of apoA-I, we utilized the apoA-I 50–75 fragment peptide, which contains two aggregation-prone residues: 53–58 and 67–72.

**Figure 8:**
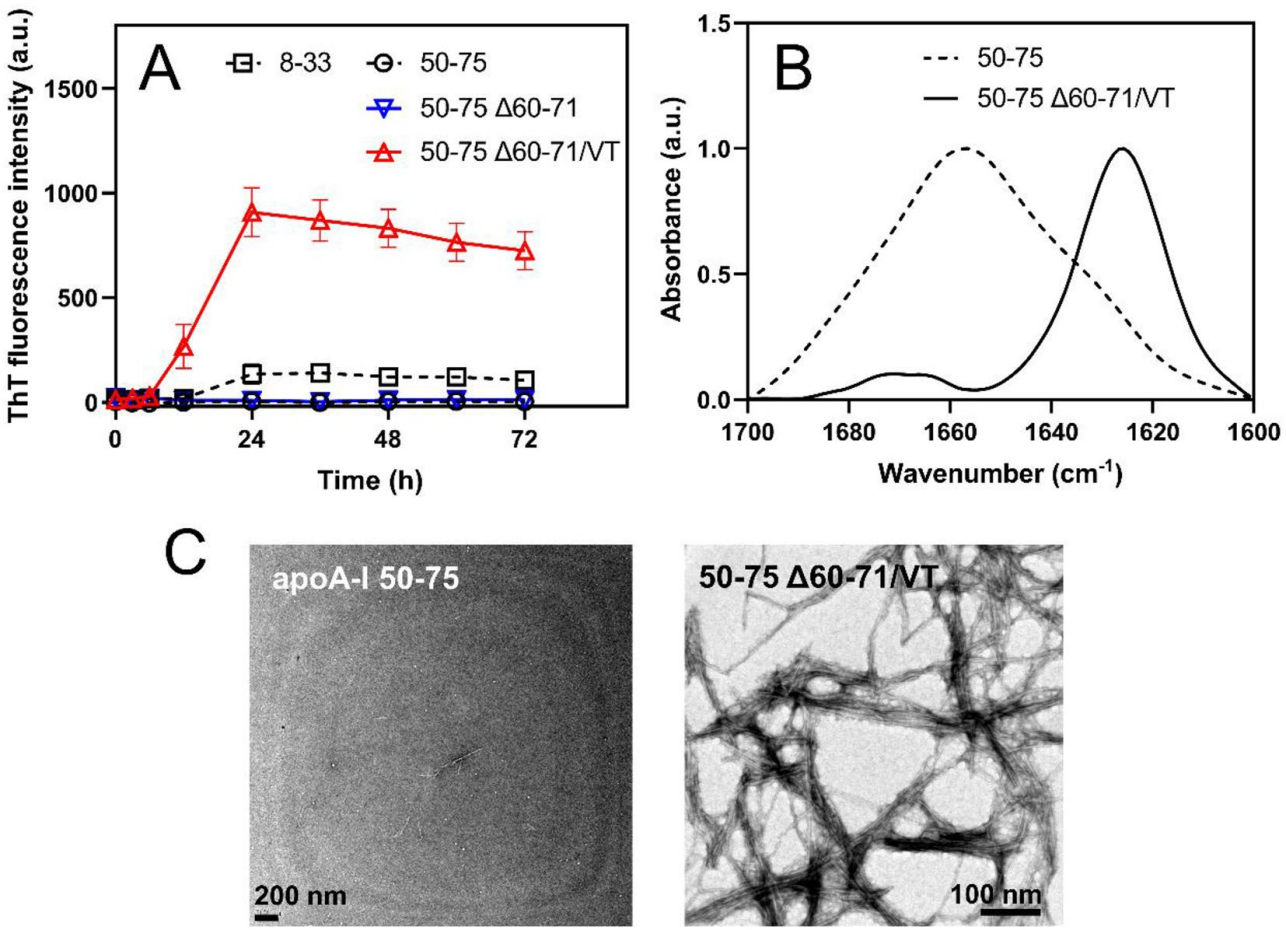
Effects of the Δ60‒71/VT mutation on the fibril-forming propensity of apoA-I 50‒75 peptide. (**A**) ThT fluorescence intensity for apoA-I 50‒75 (open circle), 50‒75 Δ60‒71(open inverse triangle), and 50‒75 Δ60‒71/VT (open triangle) peptides were plotted as a function of time. The data for the apoA-I 8‒33 peptide (open square) was shown for comparison. Peptide and ThT concentrations were 0.2 mg/ml and 10 μM, respectively. *a. u.*, arbitrary units. (**B**,**C**) ATR-FTIR spectra (**B**) and TEM images (**C**) of apoA-I 50‒75 and 50‒75 Δ60‒71/VT peptides after incubation for 72 h.

The ThT fluorescence assay revealed that the apoA-I 50–75 Δ60–71/VT peptide exhibits a strong propensity to form fibrils, while the apoA-I 50–75 and 50–75 Δ60–71 peptides demonstrate negligible fibril-forming ability under our experimental conditions. The apoA-I 8–33 peptide that contains an aggregation-prone segment of residues 14–22 exhibited a moderate property to form fibrils^43^. Consistent with these findings, attenuated total reflection Fourier-transform infrared (ATR-FTIR) spectra of the apoA-I peptides after incubation indicated that the apoA-I 50–75 Δ60–71/VT peptide exhibited a distinct band at approximately 1630 cm^–1^, whereas the dominant component for the apoA-I 50–75 peptide was approximately 1655 cm^–1^ (Fig. 8B). These spectral differences suggest that the apoA-I 50–75 Δ60–71/VT peptide undergoes a transition to a β-sheet-rich structure upon incubation. Furthermore, TEM observations revealed that the apoA-I 50–75 Δ60–71/VT peptide apparently forms straight fibrils after incubation, whereas the apoA-I 50–75 peptide does not (Fig. 8C, D). Taken together these results provide clear evidence that the Δ60–71/VT mutation greatly enhances the aggregation-prone nature of the residues 50–75 segment in the apoA-I 1–83 fragment.

## Discussion

Many human exchangeable apolipoproteins, including apoA-I, are known as amyloid precursor proteins involved in hereditary or acquired forms of disease^44,45^. These apolipoproteins have partially folded dynamic conformations in the lipid-free state. In apoA-I, the N-terminal helix bundle domain contains two major aggregation-prone segments of residues 14–22 and 53–58, as well as a minor segment of residues 69–72^18,25^. It has been proposed that amyloidogenic mutations that structurally destabilize apoA-I increase the exposure of these aggregation-prone segments, leading to protein aggregation and fibril formation^17,46^. However, the present results (Fig. 1) indicate that structural destabilization and exposure of hydrophobic surfaces in the N-terminal helix bundle alone are not sufficient to trigger fibril formation by full-length apoA-I at neutral pH, consistent with previous studies^12,47^. Other factors such as oxidative modification of methionine residues^48–50^, proteolysis^51^, or interactions with amyloid-associated proteins^52^ or heparan sulfate^53,54^ may be required to initiate the conversion of full-length apoA-I into the amyloid fibrillar form at neutral pH.

In contrast to the full-length form, the N-terminal 1–83 fragment of apoA-I, which predominantly exists as a random coil structure in solution shows a strong propensity to form amyloid fibrils at neutral pH^12^. Certain amyloidogenic mutations can enhance the aggregation and fibril formation of apoA-I 1–83 fragments in the aqueous phase^12^, on lipid membranes^43,55^, or in the presence of heparin^56^. Our recent findings demonstrated that the two major aggregation-prone segments, residues 14–22 and 53–58, play critical roles in fibril formation of the G26R variant of apoA-I 1‒83 fragment. Residues 14‒22 are necessary for β-transition and fibril formation, whereas residues 53‒58 entropically promote nucleation^25^. Consistently, these two amyloidogenic segments were found to be in close proximity, forming amyloid core structures in apoA-I 1‒83/G26R fibrils^25^.

An analysis of the dependence of fibril-forming kinetics on the monomer concentration of apoA-I 1‒83 variants reveals that saturation of the elongation step occurs at high monomer concentrations (Fig. 5). It has been suggested that, at sufficiently high protein concentrations, the rate-determining step for elongation involves a transition from the diffusive attachment of a monomer to the fibril end to the structural rearrangement of the monomer incorporated into the fibril. This transition is independent of the monomer concentration^33,37^. The parallel shifts observed in the double logarithmic plots for all apoA-I 1‒83 variants (Fig. 5C) indicate that such saturation of the elongation step occurs, despite the acceleration of the nucleation step^33,57^. Furthermore, experiments on seeded aggregation kinetics demonstrate that the length of the lag phase decreases with increasing seed concentration, without substantially affecting the fibril elongation rate (Fig. 6). This finding suggests that primary nucleation is the dominant process during the lag phase of fibril formation in apoA-I 1‒83 variants. It is possible that the relatively less hydrophobic characteristics of the apoA-I 1‒83 fragment, along with the presence of the negatively charged amino acid-rich C-terminal region^43^, favor primary nucleation over surface-catalyzed secondary nucleation processes^34,58^.

A significant finding of this study was that the Δ60–71/VT mutation, among the amyloidogenic mutations occurring near the aggregation-prone segment of residues 67–72, significantly enhanced nucleation and fibril elongation during the formation of fibrils by the apoA-I 1‒83 fragment (Fig. 2 and Supplementary Fig. S1). Consistently, we observed that the Δ60–71/VT mutation greatly enhanced the ability of the apoA-I 50–75 fragment peptide to form fibrils, in which only the Δ60–71 mutation was not enough to enhance the aggregation propensity (Fig. 8). Sequence-based analyses of amyloid propensity prediction for apoA-I 1‒83 variants (Supplementary Fig. S6) indicated that the Δ60–71/VT mutation combines the two aggregation-prone segments of residues 53–58 and 67–72 to generate a larger aggregation-prone segment encompassing residues 53–62 (VTSTFSKVTW). This newly formed aggregation-prone segment is comparable to the largest aggregation-prone segment of residues 14‒22, which is necessary for fibril formation in the apoA-I 1‒83 fragment^25^.

Thermodynamic analyses of fibril formation kinetics (Fig. 7 and Supplementary Fig. S5) gave further insights into the aggregation mechanism of apoA-I 1‒83 variants. As observed in this study (Table 2), it was reported that the activation enthalpies of nucleation and fibril elongation are unfavorable for many amyloidogenic proteins, likely because the net unfavorable formation and breakage of many weak interactions are necessary to reach the transition state^38,59,60^. Regarding the activation entropy of nucleation, in contrast, we found in the present study that the Δ60–71/VT variant exhibits an almost eliminated activation entropy for nucleation during fibril formation, unlike the large unfavorable activation entropy observed in other variants (Table 2). In the previous studies, the favorable activation entropy of nucleation was observed in the G26R variant of the apoA-I 1‒83 fragment^25^ and β-amyloid 42^38^, likely arising from the desolvation of amyloidogenic regions in the protein molecule at the transition state^25,38,60^. In this regard, desolvation of the large aggregation-prone segment around residues 53–62 in the Δ60–71/VT variant may contribute to an elimination of entropic barrier for nucleation, providing a template for the intermolecular β-sheet formation^10^.

Regarding fibril elongation, the Δ60–71/VT variant exhibits similar unfavorable activation entropy for fibril elongation to those of other apoA-I 1‒83 variants (Table 2). Since the rate of fibril formation is associated with the mechanical stability of the fibril state, faster protein aggregation likely results in a fibrillar state with higher mechanical stability^61^. Therefore, it is plausible that the increased stability of the fibrils formed by the Δ60–71/VT variant, compared to the other 1–83 variants (Fig. 4B), is linked to its faster fibril elongation. It should be noted that the enhanced stability of the Δ60–71/VT fibrils against urea-induced disaggregation may be attributed to the distinct amyloid core structures composed of highly amyloidogenic segments in the apoA-I 1‒83 fragment^25^.

In summary, we have demonstrated, for the first time, that the amyloidogenic Δ60–71/VT mutation significantly enhances the formation of fibrils in the N-terminal 1‒83 fragment of apoA-I. Specifically, the Δ60–71/VT mutation generates a large aggregation-prone segment spanning residues 53–62, which plays a crucial role in promoting the nucleation process during fibril formation. This aggregation-prone segment promotes the nucleation through reducing entropic barrier, likely serving as a template for nucleation of intermolecular aggregation. These findings emphasize the pivotal role of amyloidogenic mutations in apoA-I in the intermolecular aggregation and nucleation of its N-terminal fragment.

## Materials and methods

### Preparation of recombinant apoA-I proteins and peptides

The thioredoxin (Trx)-fused wild-type N-terminal 1–83 fragments of apoA-I and amyloidogenic Δ60–71/VT, Δ70–72, and F71Y variants expressed in *E. coli* were purified as previously described^12^. Trx was cleaved by thrombin, which produced apoA-I fragments with two extra N-terminus amino acids, Gly-Ser. The apoA-I preparations were at least 95% pure, as assessed by sodium dodecyl sulfate–polyacrylamide gel electrophoresis. The apoA-I 50‒75 (WDSVTSTFSKLREQLGPVTQEFWDNL), 50‒75 Δ60–71 (WDSVTSTFSKWDNL), and 50‒75 Δ60–71/VT (WDSVTSTFSKVTWDNL) peptides were synthesized by a solid-phase method using Fmoc chemistry as described^24^. The N- and C-termini were capped with acetyl and amide groups, respectively. All apoA-I fragments were solubilized in a 6 M GdnHCl solution, which was dialyzed into the appropriate buffer before use.

### Preparation of seed fibrils

Seed fibrils were produced by incubating 500 μg/ml of the apoA-I solution at 37 ºC with shaking, followed by centrifugation at 20,000*g* for 40 min. Fibril pellets were collected and resuspended in 10 mM Tris buffer (150 mM NaCl, 0.02% NaN_3_, pH 7.4) using a Branson bath-type sonicator for 1 min. The resultant seed fibril solution was stored at 4 °C, and continuously sonicated for 1 min before use.

### CD spectroscopy

We recorded far-UV CD spectra in the wavelength range of 190‒260 nm at 25 °C using a Jasco J-1500 spectropolarimeter (JASCO, Tokyo, Japan) as previously described^25^. The α-helix content was derived from the molar ellipticity at 222 nm ([*θ*]_222_) using the equation: % α-helix = [(–[*θ*]_222_ + 3000)/(36,000 + 3000)] × 100^62^.

### ATR-FTIR spectrometry

A Jasco FTIR spectrometer FT/IR-4700 equipped with an ATR PKM-Ge-L reflectance accessory was used to record the ATR-FTIR spectra. An aliquot of the apoA-I peptide samples (1.4 mg/ml) in Tris buffer (pH 7.4) was spread on a germanium waveguide and dried under flowing nitrogen gas. ATR-FTIR spectra in the wavenumber range of 1000–3500 cm^‒1^ were obtained at a resolution of 4 cm^‒1^ with 256 accumulations under continuous nitrogen purging.

### Fluorescence measurements

Fluorescence measurements were performed on an F-7000 fluorescence spectrophotometer (Hitachi High-Technologies, Tokyo, Japan) and an infinite 200 PRO plate reader (TECAN) at 25 °C. To monitor the exposure of hydrophobic sites on the apoA-I variants, 8-anilino-1-naphthalenesulfonic acid (ANS) fluorescence spectra were collected from 400 to 600 nm at an excitation wavelength of 395 nm in the presence of 50 μg/ml protein and an excess of ANS (250 μM). The kinetics of amyloid fibril formation was monitored by measuring the fluorescence intensities of ThT^63^. Solutions of the apoA-I 1‒83 variants or peptides (200 μg/mL) in Tris buffer (pH 7.4) were incubated and shaken at 37 ºC on a microplate shaker in the presence of 10 μM ThT. Time-dependent increases in ThT fluorescence intensity were fitted to the following sigmoidal equation^25,64^:

1 $$ F = F_{0} + \frac{{F_{\max } - F_{0} }}{{1 + \exp [k_{{{\text{app}}}} (t_{m} - t)]}} $$

where *F* is the fluorescence intensity, *F*_0_ and *F*_max_ are the initial and final baselines during the lag and plateau phases, respectively. *k*_app_ is the apparent rate constant for fibril elongation and *t*_m_ is the time to 50% of the maximal fluorescence. The lag time is given as *t*_m_ − 2/*k*.

We analyzed the ThT fluorescence data using the Finke‒Watzky equation for a two-step model of nucleation followed by autocatalytic growth^30,65^:

2 $$ \frac{{F - F_{0} }}{{F_{\max } - F_{0} }} = 1 - \frac{{k_{1} + k_{2} [{\text{A}}]_{0} }}{{k_{1} \exp (k_{1} + k_{2} [{\text{A}}]_{0} )t + k_{2} [{\text{A}}]_{0} }} $$

where [A]_0_ is the initial monomer protein concentration, and *k*_1_ and *k*_2_ are the rate constants for nucleation and fibril elongation, respectively. The thermodynamic parameters for nucleation and fibril elongation were determined using the Eyring equation:

3 $$ \ln \left( \frac{k}{T} \right) = - \frac{{\Delta H^{*} }}{R}\frac{1}{T} + \frac{{\Delta S^{*} }}{R} + \ln \left( {\frac{{k_{{\text{B}}} }}{h}} \right) $$

where *k*_B_ is the Boltzmann constant and* h* is the Planck constant. The slope and y-intercept of the linear plot according to Eq. (3) give the activation enthalpy (ΔH^*^) and entropy (ΔS^*^), respectively. The activation Gibbs free energy (ΔG^*^) was obtained from ΔH^*^ and ΔS^*^ according to ∆G^*^ = ∆H^*^ – *T*∆S^*^.

To compare the stability of the fibrils against denaturant-induced disaggregation, fibrils formed by apoA-I 1‒83 variants (50 μg/ml in Tris buffer, pH 7.4) were incubated overnight at 4 °C with various concentrations of urea in the presence of ThT (10 μM), during which ThT fluorescence intensities were monitored as described above.

### AFM

AFM was performed as previously described^25^. Briefly, samples were deposited on freshly cleaved mica, and AFM images were obtained under ambient conditions at room temperature using a NanoScope^®^ IIIa Tapping mode AFM (Veeco, Plainview, NY) and a micro cantilever OMCL-AC160TS-R3 (Olympus, Tokyo, Japan).

### TEM and TIRFM

TEM and TIRFM were performed as previously described^39,66^. Briefly, for TEM, the samples were negatively stained with a phosphomolybdic acid solution, and TEM measurements were performed using a JEOL JEM-1200EX transmission microscope (JEOL, Tokyo, Japan) at an acceleration voltage of 80 kV. ThT fluorescence images were obtained using an inverted microscope (IX70; Olympus, Tokyo, Japan). An argon laser was used to excite the ThT. The signals were cleaned using using a band-pass filter and visualized using an SIT camera equipped with an image intensifier.

### Cytotoxicity assay

The cytotoxicity of fibrils formed by apoA-I fragments against HEK293 cells was measured using a 3-(4,5-dimethylthiazol-2-yl)-2,5-diphenyltetrazolium bromide (MTT) assay, as previously described^12^. Briefly, HEK293 cells were plated and grown on poly-l-lysine-coated 24-well plates in DMEM containing 2% Fetal Bovine Serum for 24 h, after which they were cultured in the presence of fibrils formed by apoA-I 1‒83 variants in 10 mM phosphate-buffered saline (pH 7.4) for 24 h. Cell viability was quantitatively determined by optically measuring the reduction of MTT to formazan by living cells.

### Statistical analysis

We analyzed the data by means of one-way ANOVA with the Dunnett’s test or the Tukey’s multiple comparisons test. Results were considered significant at *P* < 0.05.

### Supplementary Information

Supplementary Figures.

## Data Availability

All data analyzed in this study are included in the article and the supplementary information.
