# Supplementary material for: Amyloidogenic 60–71 deletion/ValThr insertion mutation of apolipoprotein A-I generates a new aggregation-prone segment that promotes nucleation through entropic effects
Source: Sci Rep. 2023 Oct 28;13:18514. doi: 10.1038/s41598-023-45803-y (PMC10613298; doi:10.1038/s41598-023-45803-y)
Supplement: Supplementary file 1 — Supplementary Figures. [file 41598_2023_45803_MOESM1_ESM.docx]

**Supplementary Information**

**Amyloidogenic 60–71 deletion/ValThr insertion mutation of apolipoprotein A-I generates a new aggregation-prone segment that promotes nucleation through entropic effects**

**Norihiro Namba^1^, Takashi Ohgita^1^, Hiroko Tamagaki-Asahina^2^, Kazuchika Nishitsuji^3^, Toshinori Shimanouchi^4^, Takeshi Sato^2^, and Hiroyuki Saito^1*^**

^1^ Laboratory of Biophysical Chemistry, Kyoto Pharmaceutical University, 5 Misasagi-Nakauchi-cho, Yamashina-ku, Kyoto 607-8414, Japan

^2^ Division of Liberal Arts Sciences, Kyoto Pharmaceutical University, 5 Misasagi-Nakauchi-cho, Yamashina-ku, Kyoto 607-8414, Japan

^3^ Department of Biochemistry, Wakayama Medical University, 811-1 Kimiidera, Wakayama 641-8509, Japan

^4^ Graduate School of Environmental and Life Science, Okayama University, Okayama 700-8530, Japan

**This supplementary data consists of:**

Figures S1–S6

**Figure S1.** Kinetics of increase in ThT fluorescence for apoA-I 1‒83 and amyloidogenic variants at 37 °C was fitted by the Finke-Watzky equation. (**A**) apoA-I 1‒83 (○); apoA-I 1‒83 Δ60‒71/VT (Δ); apoA-I 1‒83 Δ70‒72 (▼); apoA-I 1‒83 F71Y (□). The solid lines are the fitted curves by the Finke-Watzky equation 2. **(B** and **C**) Comparison of rate constants of nucleation (*k*_1_) and fibril elongation (*k*_2_) for fibril formation of apoA-I 1‒83 variants according to the Finke-Watzky equation.

**Figure S2.** Urea-induced disaggregation of fibrils formed by apoA-I 1‒83 variants. Changes in ThT fluorescence spectra of apoA-I 1‒83 Δ70‒72 (**A**) and apoA-I 1-83 F71Y (**B**) with increasing concentrations of urea. *a. u.*, arbitrary units.

**Figure S3**. Cytotoxicity of apoA-I 1‒83 and amyloidogenic variants in the monomeric or fibrillar forms. ApoA-I 1‒83 variants before or after incubation for 120 h in phosphate-buffered saline (PBS) were added to the medium of HEK293 cells at a final concentration of 1 μM. The control is the result for cells treated with the same amount of PBS as those used for cells treated with apoA-I fibrils.


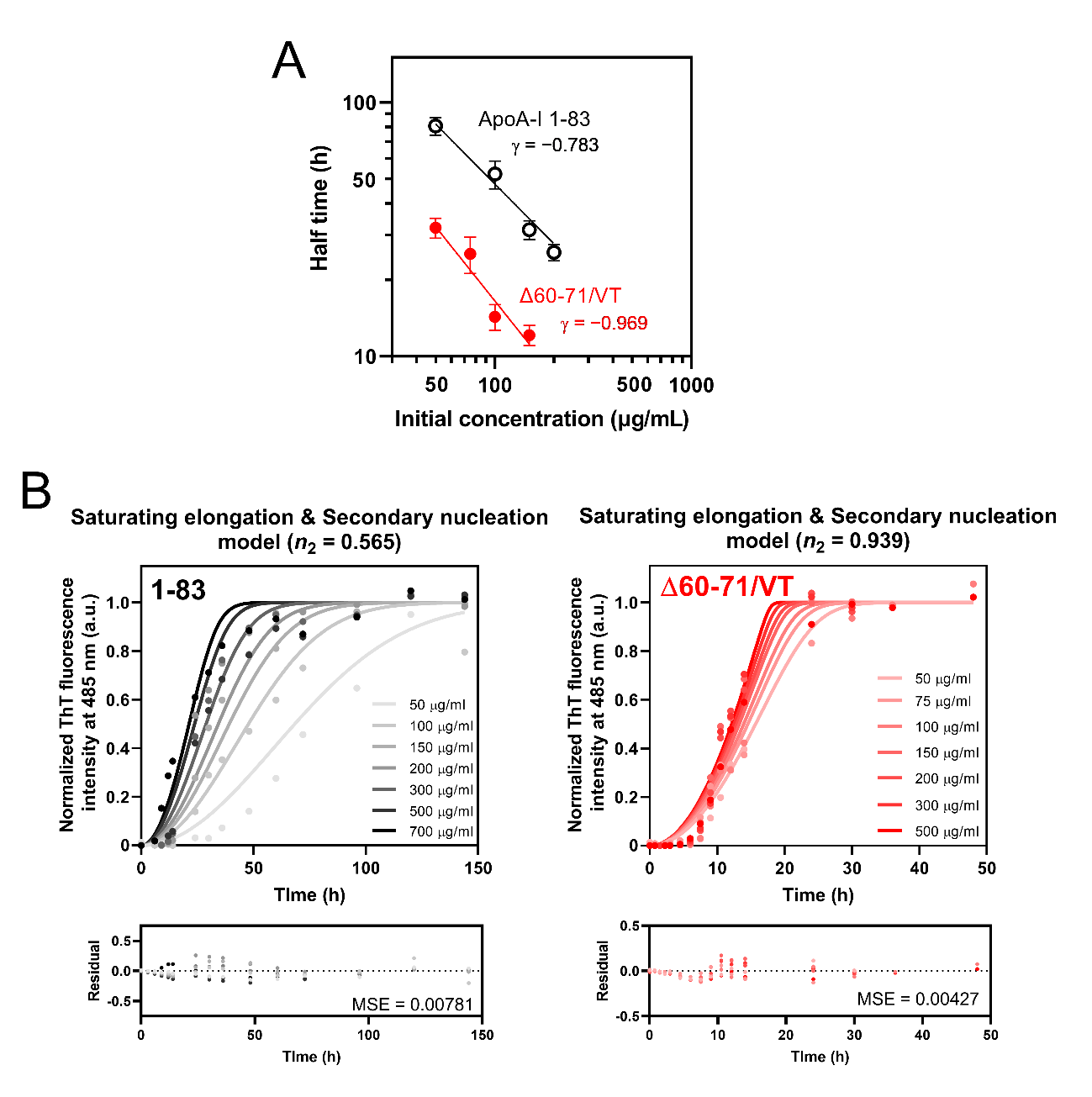


|  |  | Saturating elongation and secondary nucleation model | | | | | |
| --- | --- | --- | --- | --- | --- | --- | --- |
|  |  | *n*_c_ | *k*_n_*k*_+_ | *n*_2_ | *k*_2_*k*_+_ | *λ* | *κ* |
| ApoA-I 1-83 |  | 1.1 | 0.0048 | 0.57 | 2.3×10^‒11^ | 0.54 | 7.1×10^‒5^ |
| Δ60‒71/VT |  | 1.0 | 0.17 | 0.94 | 5.8×10^‒11^ | 2.7 | 1.2×10^‒4^ |

**Figure S4**. (**A**) Double logarithmic plot of half time and initial monomer concentration, which gives the scaling exponent, γ, as the slop of the plot. (**B**) Amylofit analysis of the kinetics data for amyloid fibril formation of apoA-I 1‒83 and apoA-I 1‒83 Δ60‒71/VT shown in Fig. 5A and 5B.

The rate constants describing primary nucleation (*k*_n_), elongation (*k*_+_), and secondary nucleation (*k*_2_) were obtained as combinations of *k*_n_*k*_+_ and *k*_2_*k*_+_ from global fitting using amyloFit (*Nat. Protoc.* 11, 252, 2016) with keeping the *n*_2_ values calculated from γ values constant. *n*_c_ and *n*_2_ are the reaction orders of primary and secondary nucleation, respectively. *λ* and *κ* are the effective noncatalytic fibril proliferation rates through primary and secondary processes, respectively, in which the relative magnitude of *λ* and *κ* determines the dominant process in the overall aggregation (*Sci. Avd.* 8, eabn6831, 2022). MSE, mean square residual error.

**F****igure S5**. (**A** and **B**) Kinetics of amyloid fibril formation monitored by ThT fluorescence for apoA-I 1‒83 Δ70‒72 (**A**) and apoA-I 1‒83 F71Y (**B**) at different temperatures. The data were from at least three independent experiments. The dashed lines are the fitted curves by the Finke-Watzky two-step model. Protein concentration was 200 μg/ml. *a. u.*, arbitrary units. (**C** and **D**) Eyring plots of rate constants of *k*_1_ and *k*_2_ for fibril formation of apoA-I 1‒83 Δ70‒72 (**C**) and apoA-I 1‒83 F71Y (**D**).

**
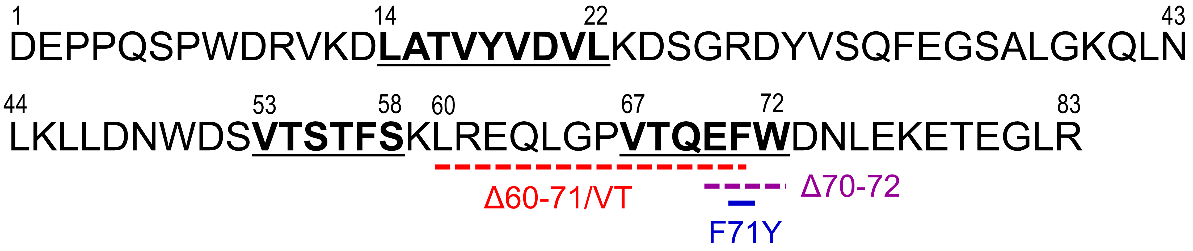
**A

**Figure S6**. (**A**) Primary sequence of the N-terminal residues 1‒83 of human apoA-I. The aggregation-prone segments (residues 14‒22, 53‒58, and 67‒72) are shown in *bold* with *underline*. Deleted or mutated regions in the amyloidogenic variants are also shown. (**B**–**E**) Amyloid propensity prediction and hydropathy of apoA-I 1‒83 and amyloidogenic variants: (**B**) apoA-I 1‒83, (**C**) apoA-I 1‒83 Δ60‒71/VT, (**D**) apoA-I 1‒83 Δ70‒72, (**E**) apoA-I 1‒83 F71Y. Amyloid propensity prediction was generated using the consensus algorithm AmylPred2 (*PLoS One* 8, e54175, 2013). Hydropathy was calculated as described by Kyte and Doolittle (*J. Mol. Biol.* 157, 105-132, 1982) using a sliding window of nine residues.
